# The Traditional Chinese Medicine MLC901 inhibits inflammation processes after focal cerebral ischemia

**DOI:** 10.1038/s41598-018-36138-0

**Published:** 2018-12-24

**Authors:** C. Widmann, C. Gandin, A. Petit-Paitel, M. Lazdunski, C. Heurteaux

**Affiliations:** Université Côte d’Azur, France; Centre National de la Recherche Scientifique (CNRS), Institut de Pharmacologie Moléculaire et Cellulaire, UMR 7275, 06560 Valbonne, France

## Abstract

Inflammation is considered as a major contributor to brain injury following cerebral ischemia. The therapeutic potential of both MLC601/MLC901, which are herbal extract preparations derived from Chinese Medicine, has been reported both in advanced stroke clinical trials and also in animal and cellular models. The aim of this study was to investigate the effects of MLC901 on the different steps of post-ischemic inflammation in focal ischemia in mice. *In vivo* injury was induced by 60 minutes of middle cerebral artery occlusion (MCAO) followed by reperfusion. MLC901 was administered in post-treatment 90 min after the onset of ischemia and once a day during reperfusion. MLC901 treatment resulted in a reduction in infarct volume, a decrease of Blood Brain Barrier leakage and brain swelling, an improvement in neurological scores and a reduction of mortality rate at 24 hours after MCAO. These beneficial effects of MLC901 were accompanied by an inhibition of astrocytes and microglia/macrophage activation, a drastically decreased neutrophil invasion into the ischemic brain as well as by a negative regulation of pro-inflammatory mediator expression (cytokines, chemokines, matrix metalloproteinases). MLC901 significantly inhibited the expression of Prx6 as well as the transcriptional activity of NFκB and the activation of Toll-like receptor 4 (TLR4) signaling, an important pathway in the immune response in the ischemic brain. MLC901 effects on the neuroinflammation cascade induced by cerebral ischemia probably contribute, in a very significant way, in its potential therapeutic value.

## Introduction

The focal hypoperfusion of the brain induced by ischemic stroke leads to excitotoxic cell death, to oxidative damage and to inflammation^1^. Inflammatory processes are present at all steps of the ischemic cascade and participate to both stroke-induced brain damage and to brain repair^2^.

Due to the disruption of the blood-brain-barrier (BBB), the recruitment and the infiltration of circulating leukocytes (neutrophils, lymphocytes and monocytes) in the ischemic brain are central events of post-ischemic inflammation. On the other hand, microglia or resident brain macrophages are the major inflammatory cell types in the CNS. Activation of microglia takes place after ischemic stroke and peaks at 2–3 days. The process lasts for weeks after the initial injury^3^. The ischemia-induced activation of both microglia and astocytes^2^ leads to release of pro-inflammatory cytokines such as interleukin 1 β (IL-1β) and tumor necrosis factor α (TNFα)^4–6^ as well as of chemokines^7,8^. These pro-inflammatory mediators facilitate BBB damage that favors the migration of circulating leukocytes into the ischemic brain^3^. However, both microglia and astrocytes have a dual function in ischemic stroke. Microglia can also produce TGF-β1, which has a neuroprotective role and astrocytes can induce the production of a number of molecules which activate anti-inflammatory responses^9^.

Cerebral ischemia triggers activation of Toll-like receptors (TLR)^2^, via endogenous ligands^2^ of the danger-associated molecular patterns (DAMPS), that are released from ischemic tissues^10^. TLR4 seems to be particularly associated with ischemic stroke^11^. Among the DAMPS released after stroke, there are members of the peroxiredoxin (Prx) family, and particularly Prx5 and Prx6 (increased 12–24 h after stroke onset). Both Prx5 and Prx6 induce activation of infiltrating macrophages and of inflammatory cytokines from invading T lymphocytes^12^. Ischemia-induced TLR4 signaling leads to activation of the transcription factor NF-κB and subsequent transcriptional induction of pro-inflammatory genes, such as cellular adhesion molecules, growth factors, matrix metalloproteinases (MMPs) and cytokines from immune cells, resulting in potent post-ischemic neuroinflammation and brain injury^10^.

The inflammatory process linked to brain ischemia is definitively complex but now well explored and provides new potential targets for future treatments for a brain disease that is very much at present in search of novel therapeutic approaches.

The recent failure of so many “classical” therapeutic strategies for stroke patients, despite promising preclinical data^13–15^, has encouraged a search on the potential therapeutic effects of cocktails of extracts of natural plants which are the active constituents of Traditional Chinese Medicine (TCM). This “medicine” has been playing an important role in health protection and disease control for thousands of years in Asia. Its potential therapeutic efficacy is usually attributed to the synergistic property of multiple herbal constituents that provide a combinational therapeutic strategy that improves the efficacy through hitting multiple targets. In the field of stroke, MLC601 (extracts of 14 natural ingredients) and MLC901 (extracts of 9 herbal components) have recently emerged as a promising treatment for improving functional recovery of patients after ischemic stroke^16–27^. The CHInese Medicine neuroaid Efficacy on Stroke recovery (CHIMES) trial in patients with stroke of intermediate severity reported that MLC601 is a safe treatment that reduces the early recurrent vascular events and vascular deaths in post-stroke patients^17,28–31^. In the Philippines cohort of the CHIMES trial (378 patients), which included more patients with predictors of poorer prognosis, MLC601 improves their functional recovery^22^. A recent extension study of the CHIMES trial (CHIMES-E) revealed that a 3-month treatment with MLC601 improved the functional outcome for up to 2 years among patients with stroke of intermediate severity^22,32^. All these clinical results are encouraging to pursue further exploration of MLC601 and MLC901 as a new efficient therapeutic strategy against stroke. Clinical trials have been successfully carried out with MLC901 for patients with traumatic brain injury^33,34^ and are ongoing for vascular cognitive impairment (Neurites)^35^. Consistent with clinical observations of the benefit of this TCM in humans, MLC601 and MLC901 have been extensively studied *in vitro* and *in vivo* using rodent models of focal and global ischemia as well as of traumatic brain injury^36–40^. The neuroprotective and neuroregenerative properties of the two mixtures have been reported to be comparable^36^. These preclinical studies have pointed out a marked beneficial effect of MLC901 on activation of ATP-sensitive potassium channels^41^, a process known to be neuroprotective against stroke^42^ as well on the repair process including neurogenesis, neurite outgrowth and angiogenesis^36,43^.

Because inflammation and its signaling pathways are considered to play key roles in the ischemic cascade as well as in the repair process, this study investigates the potential anti-inflammatory effects of MLC901 in a model of transient focal cerebral ischemia in mice, and analyzes the effect of the TCM on the different mechanisms that trigger the inflammatory process.

## Results

### MLC901 protects ischemia-induced brain damage, BBB breakdown, cerebral edema and neurological dysfunction

**Figure 1:**
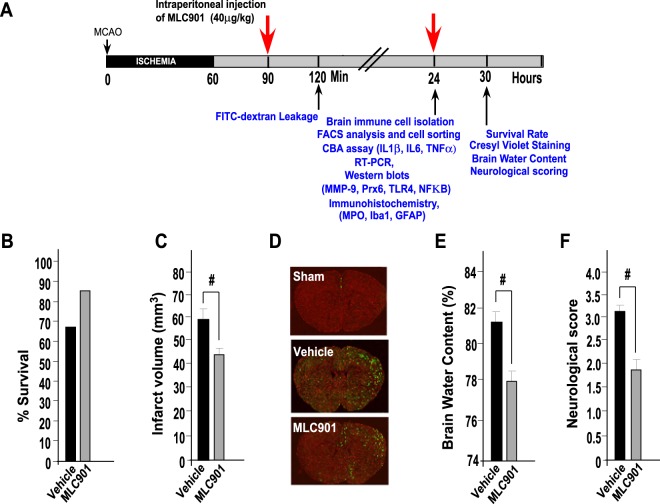
MLC901 reduced mortality rate, cerebral infarct volume, BBB leakage, brain edema and neurologic deficits induced by focal 60-min ischemia. (**A**) Flowchart illustrating the experimental design. (**B**) Percentage of survival rate and (**C**) Infarct volume (mm^3^) measured 30 hours post-ischemia (n = 10 per experimental group). (**D**) Representative images of FITC-dextran leakage (in green) in brain sections of mice submitted to focal ischemia and observed 120 min after MCAO (n = 4 per experimental group). (**E**) Percentage of brain water content in ipsilateral cortical segments measured 30 hours post-ischemia. (n = 6 per experimental group,). (**F**) Neurological score 30 hours post-ischemia (n = 10 per experimental group). MLC901 was intraperitoneally injected with a single dose of MLC901 (40 μg/kg) diluted in saline (as vehicle) 30 min after the onset of ischemia. Data are reported as mean ± SEM. ^#^*P* < 0.05 *versus* vehicle ischemic group (Mann & Whitney test).

The flowchart illustrating the experimental design is shown in Fig. 1A. To confirm the protective effects of MLC901 with the dose and the protocol used in this work, survival rate, cerebral infarction, BBB disruption, cerebral edema and neurological dysfunction induced by 60 min MCAO were evaluated 30 hours following stroke, according to our previous works on focal ischemia^36,43^. The mortality rate was reduced in MLC901-treated mice compared to vehicle-treated animals (Fig. 1B, 66.7% survival in MLC901 group as compared to 83.3% survival in the vehicle group,). MLC901 also attenuated markedly the infarction size produced by 60 min of ischemia (Fig. 1C; 43.9 ± 0.2 mm^3^ in the MLC901 group versus 59.4 ± 0.3 mm^3^ in the vehicle group, ^#^*P* < 0.05). MLC901 strongly reduced the early BBB leakage (Fig. 1D and reduced brain edema (Fig. 1E). The FITC-dextran staining appeared inhomogeneous in cortical and subcortical regions, reflecting probably the regular presence of ‘mini cores and penumbras’ in the ischemia-affected area^44^. These results fully confirm previous observations on the decrease of cerebral infarction induced by a MLC901 treatment^36,43,45^.

The neurological outcome was also improved by MLC901. Ischemia-induced neurological deficits, assessed by neurological Bederson scores, were significantly attenuated in MLC901-treated mice 30 hours after MCAO (Fig. 1F, 1.8 ± 0.5 *versus* 3.15 ± 0.2 in the vehicle group, ^#^*P* < 0.05) suggesting a better functional recovery of animals. This result is in line with previous data from our laboratory showing MLC901 efficacy to decrease the impact of stroke on neurological deficits by using a variety of behavioral tests including rotarod, pole test, grip test, Morris water maze test^37,38,43^ in addition to Bederson score^36^.

### MLC901 decreased the stroke-induced pro-inflammatory infiltration of phagocytes

**Figure 2:**
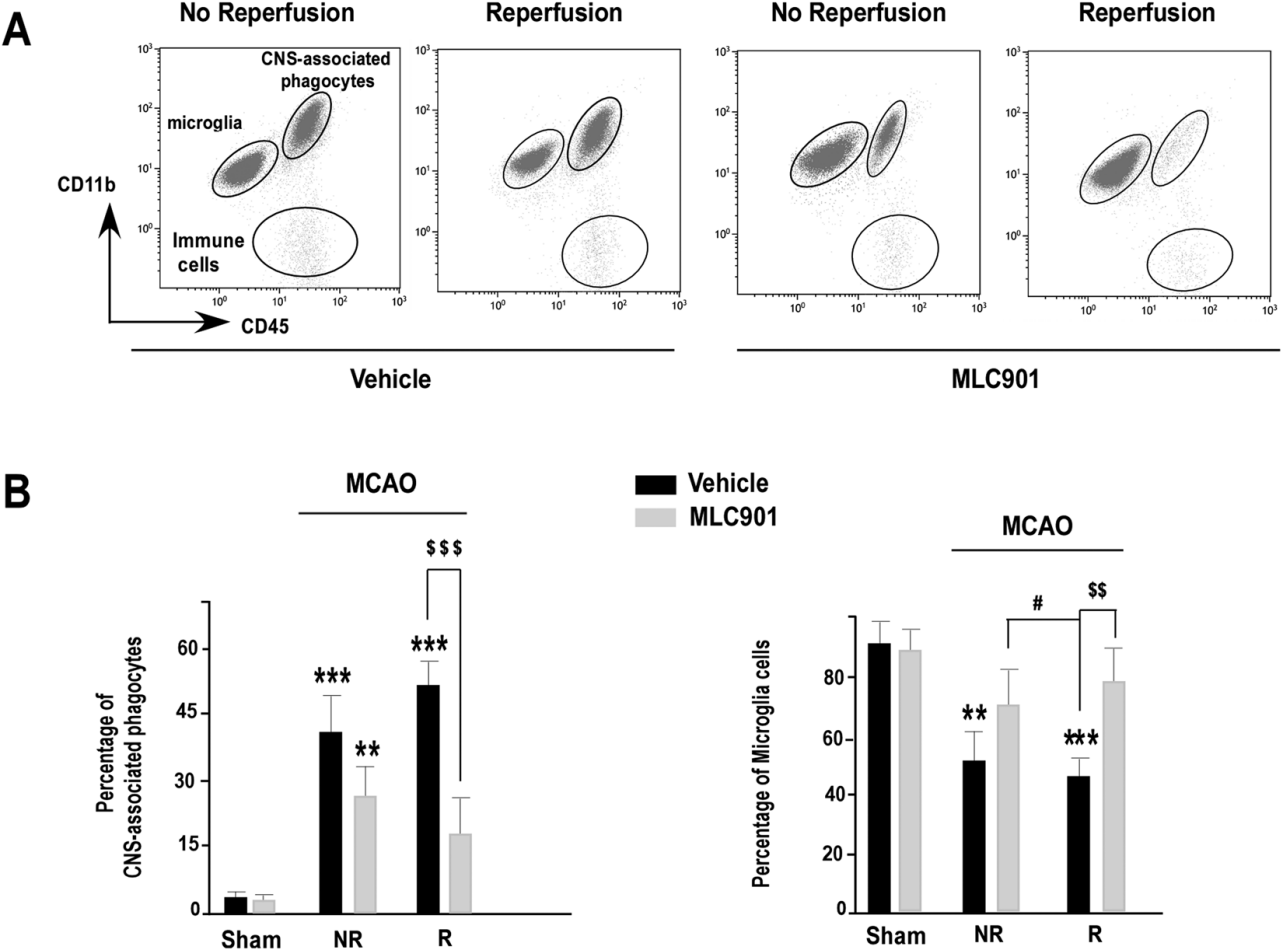
MLC901 reduced the increase in CNS-associated phagocytes induced by focal 60-min ischemia in mouse brains. (**A**) Representative bivariate dot plots of Percoll isolated brain cells illustrating on the identification of microglia (CD11b^+^/CD45^low+^), CNS-associated phagocytes (CD11b^+^/CD45^high+^) and immune cells (CD11b^−^/CD45^high+^). (**B**) Histograms of average percentage of CNS- associated phagocytes (left) and microglia (right) in live single immune cells from reperfused (R) or not reperfused (NR) brains of mice submitted to 60-min focal ischemia and treated with vehicle (black bars) or MLC901 (*ip* injection, 40 μg/kg) (n = 6 to 10 per experimental group) and sacrificed 24 hours after MCAO. Data are reported as mean ± SEM. **P < 0.01, ***P < 0.001 versus sham-operated group and ^$$^*P* < 0.01, ^$$$^*P* < 0.001 versus vehicle ischemic group, #P < 0.05 versus non-reperfused MLC901-group (Kruskal-Wallis test, H_(4,54)_ = 35.39).

Brain ischemia induces an early microglial activation and an infiltration of circulating leukocytes to the brain^2,3,46^. Using specific surface marker labeling coupled to a detailed flow cytometry analysis at 24 hours post-ischemia, we were able to distinguish CNS-inflammatory phagocytes and microglia based on differential antigen expression in ischemic brains^47^. Microglia and phagocytes were essentially identified based on their CD11b and CD45 expression levels. Microglia express high levels of CD11b and low levels of CD45 (CD11b^+^/CD45low^+^) while the heterogeneous population of CNS-associated phagocytes including brain macrophages express high levels of both CD11b and CD45 (CD11b^+^/CD45high^+^)^47^. With the CD11b and CD45 labeling (Fig. 2A), three different populations were identified in the ischemic brains: CD11b^+^/CD45high^+^ (CNS-associated phagocytes), CD11b^+^/CD45low^+^ (microglia), and CD11b^−^/CD45 high^+^ cells (immune cells) and their number changed in function of treatment and reperfusion. Figure 2A,B shows the results obtained at 24 hours following stroke. Compared to the sham group, MCAO induced a strong increase of CNS-associated phagocytes both in saline- and MLC901-treated mice (***P < 0.01 *versus* the sham group). Reperfusion alone did not change the number of CNS-associated phagocytes in vehicle-treated mice. However, compared to vehicle, MLC901 induced a significant decrease of phagocytes infiltration for animals which have reperfused (^$$$^*P* < 0.001 *versus* the vehicle-treated group) (Fig. 2B). MLC901 also preserved the normal pool of microglial cells that was strongly decreased after MCAO in vehicle-treated mice. The number of microglia cells was close to that of sham animals in the MLC901-treated group. There was no effect of reperfusion but a strong effect of MLC901.

**Figure 3:**
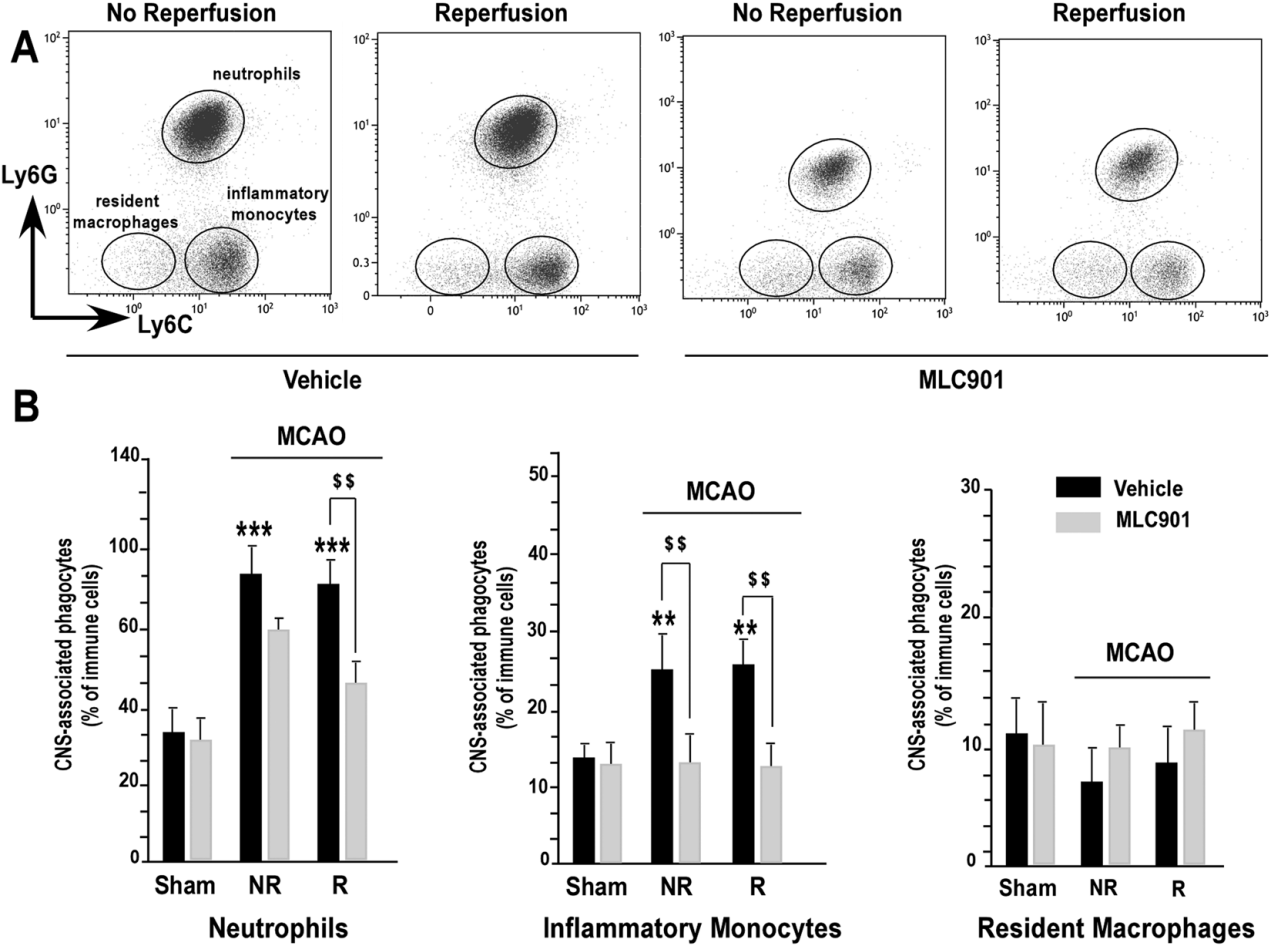
MLC901 reduced the infiltration of neutrophils and inflammatory monocytes induced by focal 60-min ischemia in mouse brains. (**A**) Representative bivariate dot plots of CNS-phagocytes stained for Ly6G and Ly6C illustrating a gating strategy to identify Ly6C^intermediate+^/Ly6G^high+^ neutrophils, Ly6C^−^/Ly6G^−^ resident macrophages and Ly6C^high+^/Ly6G^−^ inflammatory monocytes. (**B**) Histograms of average percentage of neutrophils (left), inflammatory monocytes (middle) and resident macrophages (right) in CNS-associated phagocyte cell population from reperfused (R) or not reperfused (NR) brains of mice submitted to 60-min focal ischemia and treated with vehicle (black bars) or MLC901 (*ip* injection, 40 μg/kg) (n = 6 to 10 per experimental group) and sacrificed 24 hours after MCAO. Data are reported as mean ± SEM. **P < 0.01, ***P < 0.001 versus sham-operated group and ^$$^*P* < 0.01 versus vehicle ischemic group (Kruskal-Wallis test, H_(4,56)_ = 23.16).

To further characterize which cell types migrate from periphery to the brain and the effect of MLC901, we performed a detailed analysis of CD11b^+^/CD45 high^+^ cells by flow cytometry^47^. Ly6C and Ly6G surface marker labeling allowed us to discriminate between three subpopulations among immune brain CD11b^+^/CD45high^+^ cells (phagocytes). Neutrophils were identified as CD11b^+^/CD45 high^+^/Ly6C intermediate^+^/Ly6G high^+^ cells and macrophages were identified as CD11b^+^/CD45 high^+^/Ly6G neg^−^ cells^47^. Among them, resident macrophages were identified as Ly6C neg- and inflammatory monocytes as Ly6C high^+^ cells (Fig. 3A). Neutrophils are the first leukocyte subpopulation to be recruited to the ischemic brain. As compared to the sham group, an extensive and significant infiltration of neutrophils was observed 24 hours after MCAO in the vehicle group (Fig. 3B, ^***^P < 0.001). Reperfusion alone had no effect. Interestingly, MLC901 strongly decreased the percentage of neutrophils, particularly when there was reperfusion (Fig. 3B, left panel). The percentage of inflammatory monocytes increased in the vehicle-treated mice as compared to values observed in sham animals (^**^P < 0.01). Reperfusion alone had no influence on the percentage of inflammatory monocytes whatever the group. MLC901 completely prevented the selective migration of inflammatory monocytes to the brain (^$$^*P* < 0.01). The percentage of resident macrophage was not affected by MCAO. Neither reperfusion alone nor MLC901 had any effect on the proliferation of resident macrophages (Fig. 3B, right panel). These results, in fact, do not exclude the possibility of a proliferation of resident macrophages induced by MCAO, but it could be that the effect, if it exists, is in fact masked by the dramatic infiltration of neurophils in the brain following stroke or else be delayed in time^47^.

The brain infiltration of lymphocytes 24 hours after MCAO has also been analyzed. Among the CD11b^−^/CD45 high^+^ population, we compared CD3−/B220+ (B lymphocytes), B220−/CD3+/CD4+ expressing cells (CD4+ lymphocytes) and B220−/CD3+/CD8+ (CD8+ lymphocytes) expressing cells^47^ in ischemic brains from vehicle- and MLC901-treated mice. While we observed no statistical difference for B-lymphocytes, we detected a significant infiltration of T- lymphocytes. There was no effect of reperfusion or MLC901 treatment at this time point (data not shown).

**Figure 4:**
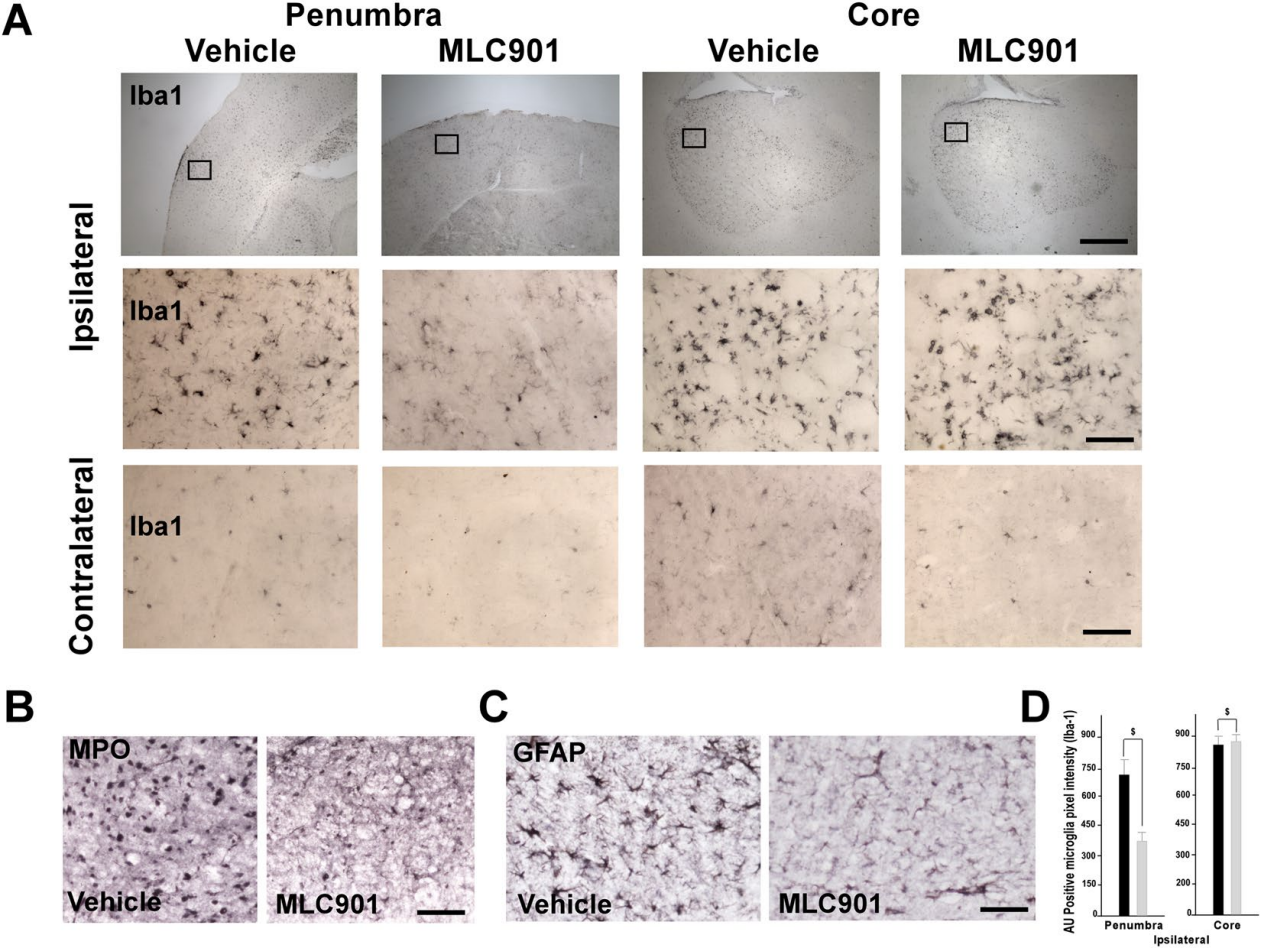
MLC901 decreased microglial activation and neutrophil recruitment in the reperfused brain 24 hours following MCAO. (**A–D**) Representative immunohistochemical staining for Iba1 microglia marker (**A**) myeloperoxidase (MPO) (**B**) and GFAP astrocytic marker (**C**) in brains of vehicle- and MLC901-treated mice 24 hours after MCAO. (**A**) Representative views of Iba1 immunopositive cells in ischemic penumbra, ischemic core and contralateral control side of vehicle- and MLC901-treated mice. Diagram boxes indicate the regions where the images in middle and bottom panels at higher magnification were acquired (**B**,**C**) Representative immunostaining of MPO (**B**) and GFAP (**C**) in ischemic penumbra of vehicle- and MLC901-treated mice. (**D**) Quantitation of Iba1 expression (AU positive microglia pixel intensity) in ischemic penumbra and ischemic core of vehicle- and MLC901-treated mice. Scale bar, 20 μm (top panels), 50 μm (middle and bottom panels) (n = 6 per experimental group).

To further analyze neutrophil infiltration to the ischemic brain we used myeloperoxidase (MPO) as biomarker. MPO is a pro-oxidative and pro-inflammatory enzyme, mainly released by activated neutrophils^48^. One day after MCAO, immunohistochemical MPO staining showed that MPO-positive cells were predominantly detected in the penumbra as previously reported^46^. MLC901 significantly reduced MPO staining, an indication of reduced neutrophil infiltration (Fig. 4B, 115 ± 41 in MLC901 group versus 148 ± 38 AU pixel intensity in vehicle group, *P* < 0.05 as compared to the vehicle-treated group). Glial fibrillary acid protein (GFAP) is a biomarker for astrocyte activation. One day after MCAO, GFAP was highly expressed in the peri-ischemic zone of the vehicle-treated animals (Fig. 4C). Treatment with MLC901 resulted in a significant decrease in the number of activated astrocytes compared with vehicle-treated ischemic mice (380 ± 42 in MLC901 group versus 770 ± 58 AU pixel intensity, *P* < 0.05, Mann & Whitney test, Fig. 4C).

Iba-1 is specifically expressed in microglia/macrophages and is up-regulated during activation of these cells by ischemia i.e. when they switch morphologically from a ramified type with retracted processes extending from the cell perikarya to an activated amoeboid form^49^. One day after MCAO, Iba1 expression in microglia was strongly increased in both penumbra and ischemic core regions of vehicle-treated mice (Fig. 4A,D) as compared to contralateral control (Fig. 4A,D). In the ischemic core area of the vehicle group, the Iba1-positive cells were a mixing of ramified and amoeboid cells, while most of Iba1 cells were ramified in the penumbra (Fig. 4A). In the penumbra, the MLC901-treatment resulted in a significant decrease in the number of Iba1-positive cells when compared with the ischemic vehicle group (Fig. 4A,D). However, MLC901 induced no significant effect on microglia activation in the ischemic core (Fig. 4A,D).

### MLC901 decreased the expression of pro-inflammatory mediators induced by stroke

**Figure 5:**
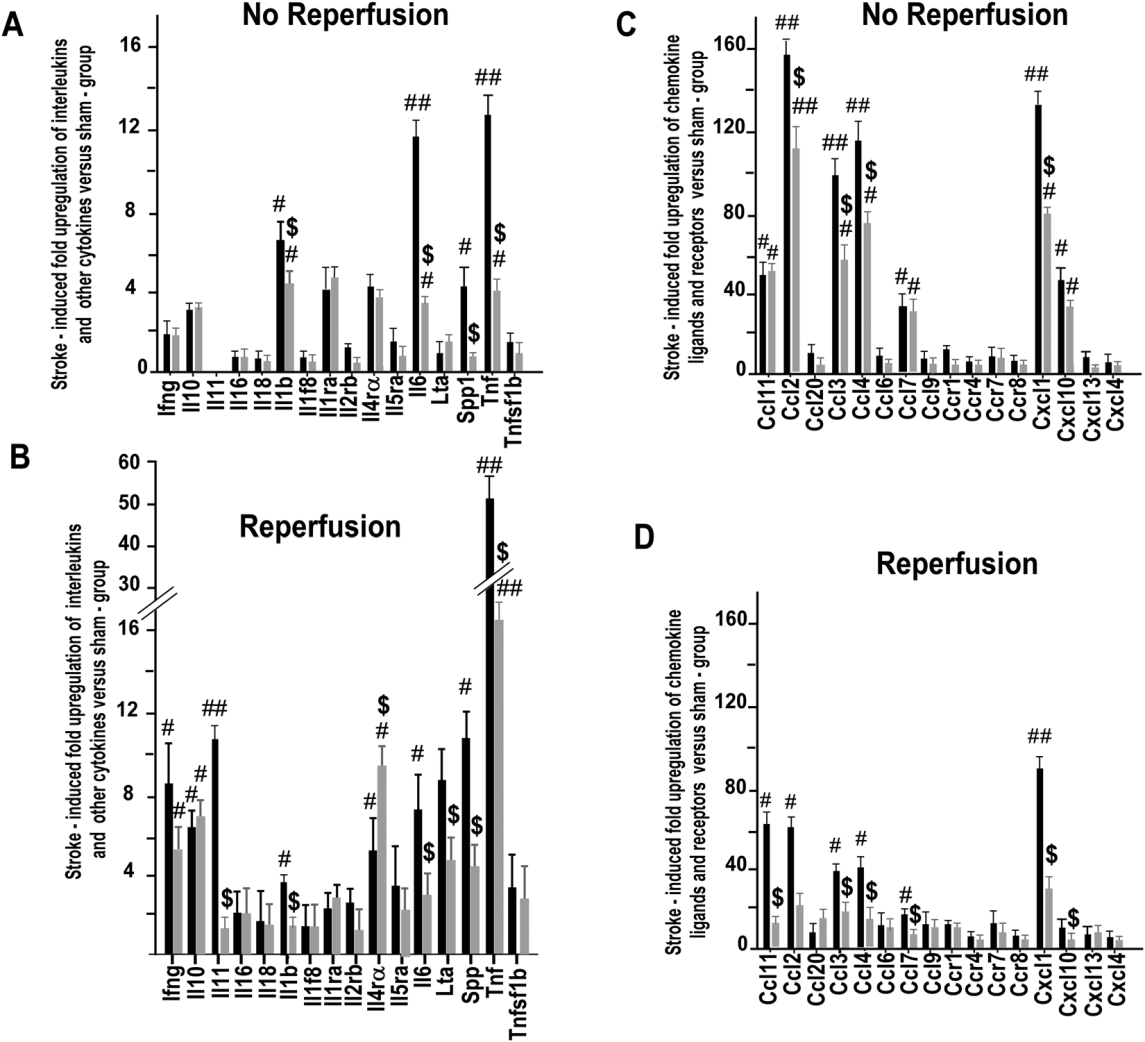
Analysis of the effect of MLC901 treatment in inflammatory marker gene expression in mouse brains after MCAO. Arrays of interleukin, cytokine (A,B, left panels) and CC chemokine ligand and receptor (A, B, right panels) gene expression in the injuried cortex of vehicle- and MLC901-treated mice 24 hours after MCAO with (**A**,**C**) or without reperfusion (**B**,**D**) (n = 6 per experimental group). MCAO-induced fold upregulation versus sham group was calculated using the ∆∆C_T_ method according to the manufacturer protocol. Data are expressed as mean ± SEM. ^#^*P* < 0.05, ^##^*P* < 0.01 versus sham and ^$^*P* < 0.05 versus vehicle-group (n = 5 per experimental group) (Mann & Whitney test).

Then, it became important to analyze whether MLC901 was able to alter the gene expression of different cytokines and chemokines induced by ischemia (Fig. 5). In the vehicle-treated group, stroke induced a many-fold increase of levels of M1-type^50^ pro-inflammatory mRNA-encoding cytokines, particularly IL11 (in the reperfused group, only), IL1β, IL6, and TNFα (in reperfused and not reperfused groups) as well as the levels of chemokines such as CCL2, CCL3, CCL4, CCL7, CCL11 and CXCL1 (in both groups). Reperfusion, in this case, played a key role on the reduction of M1-type pro-inflammatory cytokines and chemokines, particularly for the efficiency of MLC901. MLC901 decreased the IL-11, IL-1β, IL-6 and TNFα mRNA levels by 93.2, 43.1, 74.9 and 75.5% respectively, in the animals with reperfusion *versus* 43.9, 34.3, 60.7, and 68.7% in the group without reperfusion (Fig. 5A–C). Interestingly, MLC901 also increased the levels of ILR-4Rα expression, a specific marker of the anti-inflammatory M2 state^50^. The same observation was made for chemokines. For example, the transcriptional levels of CCL11, CCL2, CCL7 and CXCL1 was decreased by 10.0-, 3.2-, 3.4- and 3.2-folds, respectively in the reperfused group *versus* 1.2-, 1.4-, 1.1 and 1.6-fold in the non-reperfused mice (Fig. 5B–D). Reperfusion induced a 6.5-fold up-regulation of the anti-inflammatory cytokine, IL10, but in this case, MLC901 had no further effect on this cytokine expression.

**Figure 6:**
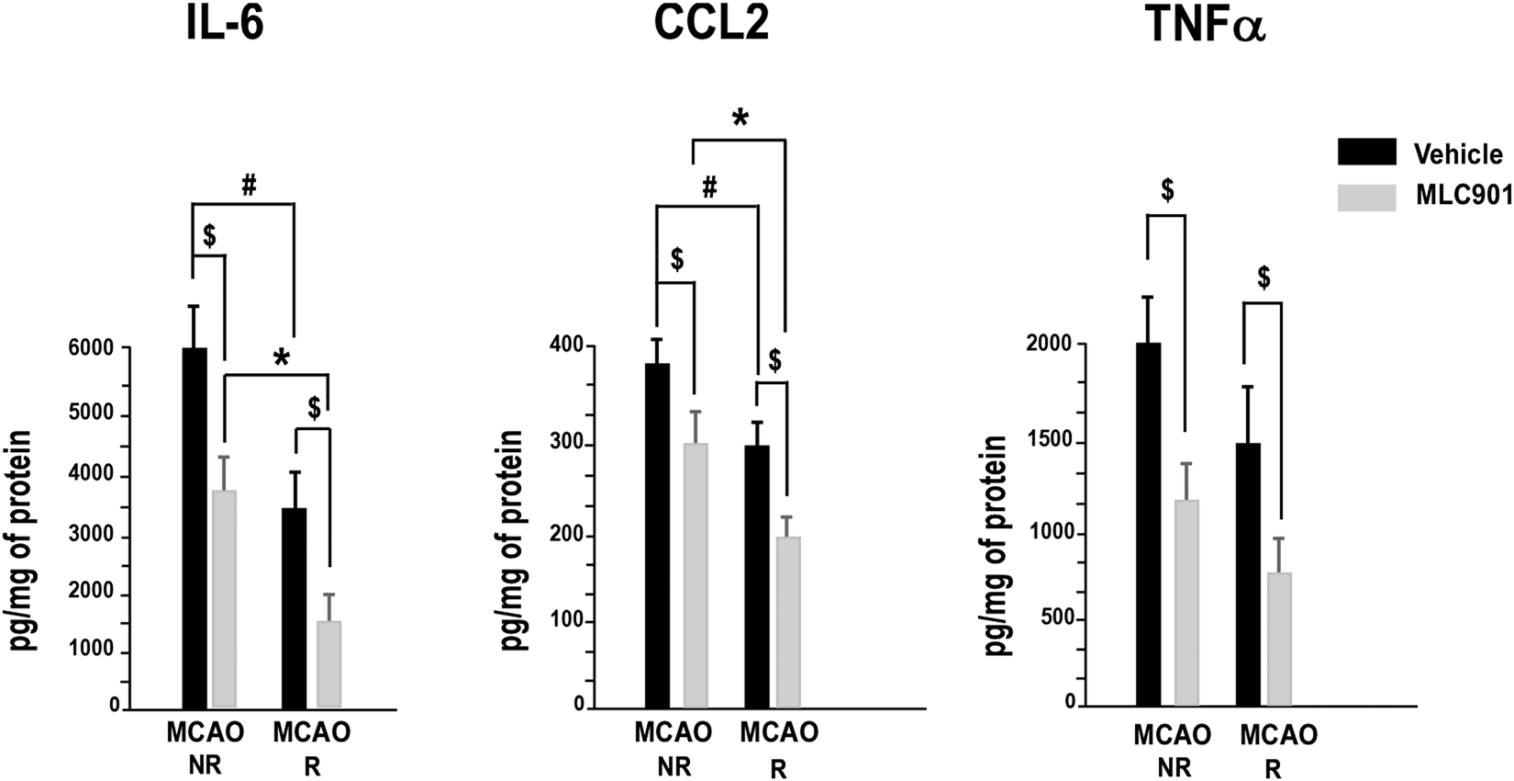
MLC901 decreased the increase of pro-inflammatory cytokine/chemokine protein concentration in mouse brains 24 hours after MCAO. The pro-inflammatory cytokines IL6, TNFα and chemokine CCL2 were quantified by cytometric bead array. Data were obtained from reperfused (R) and not reperfused (NR) brains. Bars represent the mean ± SEM. ^$^*P* < 0.05 versus vehicle-group, ^#^*P* < 0.0 versus non-reperfused vehicle-group, *P < non-reperfused MLC901-group (n = 6 to 10 per experimental group) (Mann & Whitney test).

The protein expression of M1-type pro-inflammatory cytokines/chemokines in the ischemic brains from vehicle- and MLC901-treated mice 24 hours after MCAO is presented in Fig. 6. MCAO induced a large increase of IL6, CCL2 and TNFα in the vehicle-treated group, as previously observed^5^. Reperfusion, by itself, decreased significantly the elevated pro-inflammatory cytokine/chemokine protein profile (^#^*P* < 0.05 *versus* Saline-MCAO R and ^*^P < 0.05 *versus* MLC901-MCAO R). MLC901 significantly reduced the ischemia-induced activation of IL6, CCL2 and TNFα in the reperfused and non-reperfused groups (^$^*P* < 0.05 *versus* the vehicle groups (NR and R)). As previously observed for the real time PCR results, the best effect of MLC901 was obtained with reperfused animals.

**Figure 7:**
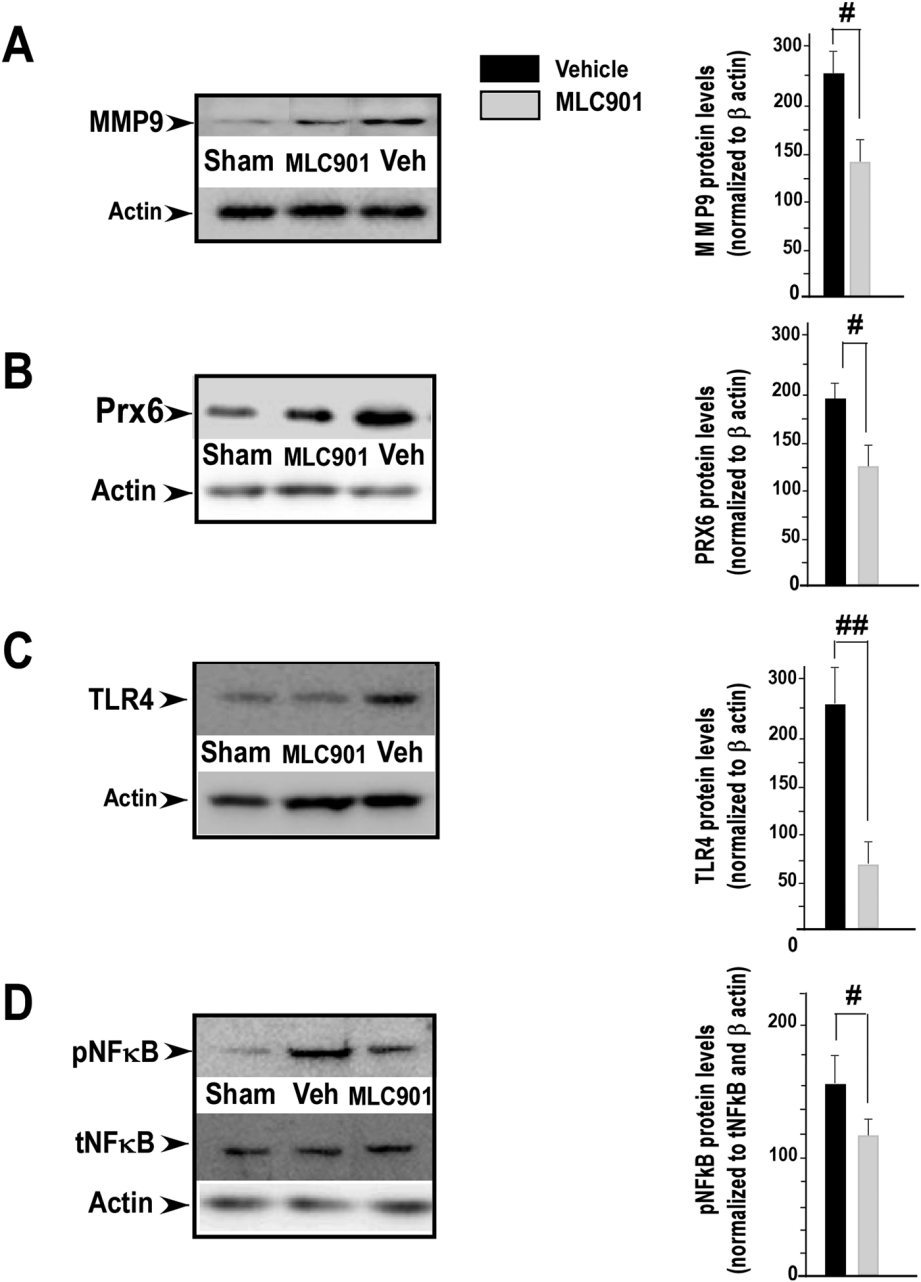
MLC901 decreased the protein expression of matrix metalloproteinase-9 (MMP-9), peroxiredoxin 6 (Prx6), Toll-like receptor 4 (TLR4) and the phosphorylation of NFκB (pNFκB) in reperfused (R) brains 24 hours after MCAO. (**A–D**) Representative images from Western blotting analysis of MMP-9 (**A**) Prx6 (**B**) TLR4 (**C**) and phosphorylated and total NFκB (left panels). Optical densitometry quantitation for MMP-9 (92 kDa), Prx6 (25 kDa), TLR4 (85 kDa) normalized to β actin and for pNFκB (65 kDa) normalized to total NFκB and β actin in injured cortical tissue (right panels). β actin was used as internal control for the loading of protein level. Data are representative of 3 separate experiments (n = 4 per group). Values (mean ± SEM) are expressed as percentage of control (^#^*P* < 0.05, ^##^*P* < 0.01 versus vehicle ischemic group) (Mann & Whitney test).

Expression and activation of matrix metalloproteases (MMP-9) following cerebral ischemia are closely associated with a disruption of BBB resulting from infiltrated peripheral neutrophils^51^. Figure 7A shows that 24 h after focal ischemia the increase of MMP-9 expression induced by MCAO in the reperfused vehicle-treated group was significantly reduced by MLC901 treatment (^#^*P* < 0.05 *versus* the reperfused vehicle group).

### MLC901 inhibits MCAO-induced expression of peroxiredoxin 6, TLR4 signaling and phosphorylation of NFkB

Peroxiredoxins (Prx’s), and particularly Prx6 are important contributors to immunomodulation and neuroinflammation after ischemic stroke^12^. As shown in Fig. 7B (left panel), the expression of Prx6 was strongly increased in the ischemic brain of reperfused vehicle-treated group compared with that observed in sham control group. MLC901 markedly decreased Prx6 expression 24 hours after MCAO (^#^*P* < 0.05 *versus* the vehicle-group, Fig. 7B right panel).

TLR4 is also related to inflammation and is associated with the pathological progression of cerebral ischemia^2,11^. As shown in Fig. 7C (left panel) and as already reported^11^, cerebral ischemia significantly induced TLR4 expression. This increased TLR4 expression did not take place in MLC901-treated mice (^##^*P* < 0.05 *versus* the vehicle group, Fig. 7C, right panel).

The NFκB signaling pathway is well-known to be associated with immune responses in the ischemic brain^52^ and NFκB activation is a major signaling system associated with TLR4^53^. Activation of NFκB was observed in response to MCAO (Fig. 7D, left panel), involving phosphorylation of the p65 NFκB subunit^53^. In the ischemic brain of vehicle-treated animals, the level of phosphorylated NFκB was strongly increased in the ischemic group as compared to the sham group, while the level of total NFκB was not changed (Fig. 7D, left panel). This increase of NFκB phosphorylation was significantly decreased by MLC901 treatment (^#^*P* < 0.05 *versus* the vehicle-treated group, Fig. 7D, right panel).

## Discussion

Accumulating evidence from animal studies^36–40,54^ and clinical trials^17,22,23,26,28,29,31,55^ indicates that MLC601 and its derivative product MLC901 probably represent a promising therapeutic strategy for brain injuries, and particularly for stroke treatment. The molecular and cellular mechanisms associated with the beneficial effects of this TCM in cellular and animal models of stroke are multifaceted. MLC901 has neuroprotective effects by acting on key players controlling the ischemic cell death. It activates ATP sensitive potassium channels (K_ATP_)^41^, a process that is neuroprotective^56,57^ and is also involved in the beneficial effects of preconditioning^57,58^. MLC901 decreases excitotoxicity^36,41^ and stimulates the Akt survival pathway. It reduces apoptosis, oxidative stress and free radical generation^37^. MLC901 is also known to improve neurorepair by stimulating BDNF production, neurogenesis, synaptogenesis and angiogenesis^36,43^.

The present work confirms that MLC901 administered 90 min after MCAO onset, and then during the entire time of recovery, significantly protects mice against brain injury after focal ischemia. MLC901 increases survival after MCAO, reduces the infarct volume and improves the neurological score. All these results are in line with previous preclinical results^36–38,45^ and also with clinical trials^17,22,23,28,29,31,55^.

The breakdown of the BBB during post-ischemic reperfusion is associated with interdependent mechanisms including inflammation, oxidative and nitrosative stress, and induction of matrix-metalloproteinases^59,60^. Preclinical studies examining BBB opening after ischemia have shown the existence of a biphasic permeability response (1–4 h leading to cytotoxic edema and 22–46 hours leading to vasogenic edema)^60,61^, while others, using MRI have found a continuous BBB leakage during one week^62^. In humans, the situation is quite comparable with a continuous BBB opening for up to 4 days after stroke^63^. In previous studies, we have reported the beneficial MLC901 effect on BBB disruption at a later stage (30 hours) both after focal ischemia^43^ and TBI^38^. In the present work, we analyzed the early BBB leakage with the FITC-dextran post-ischemia and observed that MLC901 attenuates the severe BBB breakdown as early as 3 hours post-MCAO and the subsequent hemispheric swelling.

While, in the present work, MLC901 was injected 90 min after MCAO onset, previous studies on animal models have reported a window of protection for MLC901 up to 3 h^37^, a long delay for mice and a delay which seems compatible with the clinical situation (early stroke management of patients in stroke centers and mobile stroke units). The anti-inflammatory effects of MLC901 probably persist for longer times, particularly for patients who continue to take the compound for periods as long as three months.

In the present study we extended the analysis of the potential beneficial effects of MLC901 in stroke treatment by examining more specifically the effects of MLC901 on inflammation in the MCAO model. Indeed, post-ischemic inflammation is now considered as an important potential target for therapeutic intervention^1,2^. Clinically, systemic inflammation seems to increase the risk of stroke and to be associated with a worse prognosis^64,65^. Stroke patients with systemic inflammation apparently exhibit poorer recovery^66^.

Intraluminal MCAO with thread filament used in the present work is one of the most relevant and widely used stroke models because it better mimics the pathophysiology of human stroke compared with permanent occlusion models in rats and mice. Most cases of human ischemic stroke have spontaneous or thrombolytic therapy-induced reperfusion^67^. Preclinical stroke studies have demonstrated that reperfusion represents an especially vulnerable period for the brain. It, of course, provides the important benefit of restoring blood flow to the ischemic region and, simultaneously, it induces a massive influx of activated leukocytes into the injured zone^68,69^. Reperfusion hastens inflammation^70^. This is why we have analyzed the effects of MLC901 against inflammation in different states of brain reperfusion.

Focal ischemia induces a rapid activation of resident cells (mainly microglial cells), production of proinflammatory mediators, and infiltration of circulating inflammatory cells (including neutrophils, T cells and monocytes/macrophages), into the ischemic brain tissue^3^. It is well known that inhibiting the inflammatory response decreases infarct size and improves neurological deficits in experimental stroke^2,71^, a result which is also seen in the present work.

Neutrophils are among the first cells in the blood to respond after ischemic stroke, after which they are phagocyted by microglia and macrophages^2,3,72,73^. Neutrophil infiltration is recognized as an important pathogenic factor following stroke^73,74^. In the present work, we observed a marked increase in neutrophil infiltration following the ischemic insult in the injured cortex of vehicle-treated mice at 24 hours post-ischemia. MLC901 treatment significantly decreased this neutrophil infiltration, suggesting that this TCM attenuates the neuronal damage caused by stroke, at least in part, *via* inhibition of harmful neutrophil recruitment. Several drugs targeting neutrophil recruitment such as enlimomab^75^, Hu23F2G or Leukoarrest^76^ and UK-279276^77^ were previously tested in clinical trials but have unfortunately not been successful, in part, for some of them, because of side-effects including leukopenia and immune suppression. MLC901 seems to be in a different category with very satisfactory safety properties observed in large cohorts of patients^24^ and benefits seen both for patients^17,18,22,23,28–35,55,78^ and in animal studies^36–40^. MLC901 provides an interesting therapeutic alternative for preventing neutrophil infiltration in stroke.

In addition to neutrophils, T-lymphocytes are the major leukocyte subpopulation involved in inflammatory brain damage after stroke^2^. While we observed, at 24 hours following ischemia, a significant infiltration of T - lymphocytes in the vehicle-treated group, MLC901 had no effect. There are many subtypes of T-lymphocytes and the time course of their recruitment remains largely undetermined. Earlier studies suggested that lymphocytes recruitment peaked between 3 and 7 days^79^ leading to the possibility that MLC901 could have had a positive effect on T lymphocytes accumulation, not in the first 24 hours after ischemia, but in a later phase that has not been analyzed in the present work.

Microglia/macrophages are highly plastic cells that can express two different and apparently opposite phenotypes: a cytotoxic M1 polarization associated to the release of destructive proinflammatory mediators such as pro-inflammatory cytokines, reactive oxygen species (ROS) and proteinases and a protective M2 polarization associated to tissue repair^2,50^. Therefore, a control of the balance between detrimental and beneficial microglia/macrophage responses has been considered a probable therapeutic strategy against stroke^50^. Stroke reinforces deleterious M1 polarization and decreases repairing M2 phenotype leading to the inflammatory cascade and to cell injury^80,81^. IL-4 or IL-10 knock-out mice have a worse outcome after cerebral ischemia due to an enhanced expression of M2 markers^82,83^. MLC901 significantly reduced the activation of microglia in the ischemic penumbra. We also observed that MLC901 markedly increased mRNA expression of IL-4Rα in microglia and monocytes of reperfused brains, an effect which would favor protective and repair processes. MLC901 seems to favor the shift of microglia from the M1 to the M2 phenotype. This is also indicated by its effects on cytokine and chemokine production induced by stroke. Previous studies in animal models and in stroke patients have shown that continued production of cytokines such as IFNγ, TNFα, IL11, IL-1β, IL-1ra and IL-6 induces and maintains the M1 polarization state^3,84^. This work also shows that a strong up-regulation of major cytokines (TNFα, IL-1β, IL-6, IL-11) is observed after focal ischemia. MLC901 counteracts this effect.

Ischemic stroke induces the release of different chemokines such as CCL-2, CCL-3, CCL-4, CCL7, CCL-11, CXCL-1, CXCL10, from the ischemic tissue. CCL2, which is the most up-regulated chemokine, in our experimental conditions, is known to be a critical factor regulating post-ischemic inflammation. It is involved in BBB disruption and in monocyte recruitment to the site of lesion after ischemic injury^8^. CCL2 expression appears in the ischemic cortex as early as 6 hours post-ischemia, peaks within 12 to 48 hours and remains elevated up to 5 days^8,85^. CCL2 deletion or absence of its receptor (CCR2) limits infarct size, reduces the inflammatory response, BBB permeability and brain edema formation^8,86^. Taken together these data suggest that inhibiting the CCL2/CCR2 signaling, as MLC901 does very potently (Figs 5 and 6), certainly contributes, in an important way, to brain protection and other beneficial effects of this TCM.

Activation of MMP-9 following cerebral ischemia is closely associated with BBB leakage and microglial activation, and causes severe brain edema or hemorrhagic transformation^59,87^. Post-ischemic BBB breakdown is reduced after MMP-9 inhibition and MMP-9 gene deletion^51^. During reperfusion, MMP9 released from neutrophils recruited to the injured brain exhibits pro-inflammatory effects, promotes further recruitment of neutrophils to the lesion site, and finally contributes to post-stroke neuronal damage^51^. This study demonstrates that MLC901 treatment significantly reduces the expression of MMP-9 in the ischemic brain. Inhibition of neutrophil recruitment by MLC901 could be the origin of this reduction, which certainly contributes to the protective effect of MLC901 against BBB disruption as well as edema formation.

NFκB is an oxidative stress-responsive transcription factor, and its involvement in ischemic injury is well recognized^88–90^. It is a key regulator of the inflammatory response. In the early phase of stroke, infiltrating neutrophils cause excessive production of ROS, resulting in oxidative stress in the injured brain tissue. Oxidative stress promotes the activation of NFκB, which regulates the transcriptional induction of various pro-inflammatory genes^52,53^. In the current study, we found that MLC901 significantly reduced the level of phosphorylated NFκB, which is a marker of NFκB activation^91^. Again, because high levels of ROS are well-known to be produced by recruited neutrophils^3^, the inhibition of neutrophil recruitment by MLC901 would probably lead to prevention of NFκB activation and to a decrease of inflammation *via* the NFκB pathway.

The TLR4/peroxiredoxin (Prx6) pathway plays a pivotal role in the post-ischemic inflammation^12^. Cerebral ischemia induces the release of the Prx6 and stimulates macrophage infiltration *via* TLR4^12^. TLR4-induced activation of NFκB and subsequent production of pro-inflammatory cytokines leads to brain inflammation and ischemic injury^12^. TLR4-knockout mice show indeed smaller infarct sizes and improved neurological deficits after stroke^92,93^, and in humans, TLR4 polymorphisms have been associated with ischemic stroke outcome^94,95^. This work confirms increased Prx6 expression in ischemic brain and the activation of stroke-induced TLR4 signaling, including enhanced TLR4 expression and NFκB activation. All of these events are inhibited by MLC901.

In summary, the major effects of MLC901 are the reduction of neutrophil recruitment, the reduction of microglia activation and the reduction of pro-inflammatory mediators (TNFα, IL6, IL1β, CCL2) production induced by stroke. The modulation of TLR4/Prx6 pathway by MLC901 seems to represent a key step in these anti-inflammatory effects and to contribute to its protective effects against stroke. In the past years a number of anti-inflammatory approaches targeting specific types of immune cells (neutrophils, microglia ….) have proven to be successful in animal models against stroke. However, the transfer to a clinical setting has, so far, been unsuccessful^3^. There are at least three reasons to be more optimistic with MLC901. The first one is that MLC901 has multiple anti-inflammatory effects on neutrophils, microglia, vascular endothelium and neuronal cells. The second one is that, instead of going, as usual, from the bench to the patient bed, here we started from the bed side (several clinical assays performed) and went successfully from there to the bench to discover the mechanisms that are responsible for the reported beneficial effects of MLC901 against stroke. The third one is that MLC901 is most probably a cocktail of molecules which explains the multifaceted positive effect of this TCM against the inflammatory process along with other types of positive effects such as neurorepair^36,45^.

## Methods

### Animals

Seven weeks old C57BL/6 male mice (Janvier) were housed five per cage on inversed 12 H light/dark cycle (light on at 08:00 PM) in animal facility maintained at ambient temperature of 21 ± 1 °C. They were provided with food and beverage *ad libitum*. Experiments were performed in accordance with the policies on the care and use of laboratory animals of European Community laws (directive 2010/63/EU) and approved by the French ministry of higher education and scientific research (approval number 01314.04). A particular effort was made to minimize the number of animals used per group when not needed. The researchers who carried out the ischemic surgery and all post-stroke experiments were blinded to the treatment code.

### Drug treatment

MLC901 combines 9 herbal extracts equivalent to the following composition of raw herbs per capsule: 0.80 g Radix astragali, 0.16 g Radix salvia miltiorrhizae, 0.16 g Radix paeoniae rubra, 0.16 g Rhizoma chuanxiong,0.16 g Radix angelicae sinensis, 0.16 g Carthamus tinctorius, 0.16 g Prunus persica, 0.16 g Radix polygalae, and 0.16 g Rhizoma acori tatarinowii. In these different herbs that are extracted to manufacture MLC901, there are a number of identified molecules such as tetramethylpyrazine (Rhizoma chuanxiong)^96,97^, ferulic acid (Radix angelicae sinensis, Rhizoma chuanxiong)^98^, ligustilide and butylidenephtalide (Radix angelicae sinensis, Rhizoma chuanxiong)^99,100^, astralagoside IV (Radix astragali)^101^, salvianolic acid B and tanshinone IIB (Radix salvia miltiorrhizae)^102,103^ that are known to be neurobeneficial and for some of them, anti-inflammatory. MLC901 (batch BRAINS provided by Moleac, Singapore) was intraperitoneally injected with a single dose of 40 μg/kg MLC901 diluted in saline (as vehicle) 90 min after the onset of ischemia and once a day during reperfusion until the sacrifice of animals as previously described^33^. The dose for MLC901 used in this work is the most efficient dose determined in previous studies for both cerebral ischemia and traumatic brain injury in rodents^36–38^. The animals were equally and randomly divided into five groups as follows: Control (sham-operated), Sal-MCAO-R (with reperfusion), Sal-MCAO-NR (no reperfusion), MLC901-MCAO-R and MLC901-NR.

### Ischemic stroke model (MCAO)

The ischemic stroke model (focal ischemia) was performed on seven weeks old male mice by transient (60 min) right middle cerebral artery occlusion (MCAO) and reperfusion, using a 6-0 coated filament (Doccol, Redlands, CA, USA) as previously described^36^. The regional cerebral blood flow was monitored by laser-Doppler flowmetry (Perimed, Craponne, France) to control MCAO severity and reperfusion. Animals presenting with sustained CBF reduction >70% during ischemia or a severe brain hemorrhage after MCAO were excluded from the study (<1%). Mice received MLC901 in post-treatment until the sacrifice of animals. Sham-operation was performed by inserting the thread into the common carotid artery without advancing it to occlude MCA. The animals were allowed to regain full consciousness on a nursing cage during 24 hours before returning to the home cage. The researchers who did the MCAO surgery were blinded to the treatment code.

### Neurological deficit scoring

Neurological deficits of mice were assessed 30 hours post-ischemia in a blinded fashion according to a scoring scale in a postural reflex test^104^: Grade 0 = no visible deficits; Grade 1 = forelimb flexion; Grade 2 = unidirectional circling when the animal is pulled by the tail; Grade 3 = circling and rolling movement; Grade 4 = decreased level of consciousness; Grade 5 = death.

### Measurements of infarct volumes, blood brain barrier (BBB) leakage and brain edema

To assess the infarct volume, as previously described^36^, mice were euthanized by decapitation 30 hours after reperfusion. Coronal frozen brain sections (20 μm thick) were stained using a solution of 1% cresyl violet in 0.25% acetic acid and mounted with Entellan. The striatal and cortical areas of infarction, outlined in light, were measured on each section using a computer image analysis system and corrected for brain edema according to Golanov and Reis^105^. Infarct volume, expressed in mm^3^ was calculated by a linear integration of the corrected lesions areas as previously described^36^.

The early phase of BBB breakdown BBB degeneration following MCAO was assessed 3 hours after MCAO by the extravasation of fluorescein isocyanate-conjugated dextran (FITC-dextran, 2000 Da, Sigma)^106,107^. 0.3 ml of 5% FITC-dextran (wt/vol in sterile PBS) was injected through the left femoral vein. All mice were reperfused for 10 min to allow sufficient circulation of FITC-dextran to the ischemic brain. The FITC-dextran leakage was visualized under confocal microscope at 488 nm. Mice were transcardially perfused with normal saline to wash away the remaining dye in the blood vessels. Brains were removed and coronal sections from bregma-1 to 1 mm were performed to visualize the blue staining at 30 hours after MCAO.

To measure the vasogenic edema, animals were sacrificed 30 hours after ischemia. As previously described^36^, the brains were divided into the ipsilateral hemisphere (ischemic side) and contralateral hemisphere. The ipsilateral hemisphere was weighed to obtain the wet weight and then dried at 110 °C for 24 hours. The brain water content in the ipsilateral hemisphere was calculated as follows: water content = (wet weight – dry weight)/wet weight.

### RNA isolation and Polymerase chain reaction array for cytokines, interleukins and chemokines

Total mRNA from ischemic brains was isolated according to the current Chomczynski method using Fast Prep apparatus (Q-Biogene, France). Two micrograms of total mRNAs were denatured at 65 °C for 5 min in the presence of 0.5 mM dNTP and oligodT primers (25 ng/μl, Promega, France).

RT^2^ Profiler Mouse Inflammatory Cytokines and Receptors Real time PCR arrays (PAMM-011F) (n = 6) were used to analyze the expression of a focused panel of genes. Data analysis was performed using the ∆∆C_T_ method according to the manufacturer’s protocol (SABio-Sciences/Qiagen, France). In each assay, PCR were performed in duplicate. Relative quantities of target genes were determined by comparison with results for the housekeeping gene GAPDH.

### Isolation of immune cells from mouse brains

Immune brain cells were isolated from ipsi- and contralateral brain hemispheres homogenates as previously described^47^. Mice were transcardially perfused with ice-cold PBS (pH 7.4, 1 mg/ml/EDTA). Collected brains were roughly homogenized in PBS, resuspended in PBS containing collagenase D (3 mg/ml) and incubated 30 minutes at 37 °C. Then, brain homogenates were filtered, centrifuged (10 min, 2000 rpm), washed and resuspended in 6 ml of 38% isotonic Percoll before centrifugation (20 min, 2000 rpm). Myelin and debris were discarded. Cell pellets containing brain immune cells were collected, washed and labeled for subsequent cell sorting and flow cytometry analysis.

### Brain immune cell staining, flow cytometry and cell sorting

Staining of brain immune cell surface antigens was performed as previously described^47^. Fc receptors were blocked with 2.4G2 antibody. Cells were incubated with the appropriate combination of conjugated antibodies: CD11b-PercP-Cy5.5, CD45-APC-Cy7, Ly6C-PE-Cy7, Ly6G-pacific blue, CD3-FITC, CD8-APC, major histocompatibility complex class II-Alexa700, CD80-V450, CD86-eFluor605, CD14-APC, TLR4-Alexa488, CD124/IL-R4α-Biotin and streptavidin-PE-Cy7 (BD Biosciences), CD4-Viogreen (Miltenyi Biotec), CCR2-PE (R&D Systems) or isotype control antibodies for 30 minutes. Cells were washed and resuspended in PBS containing 0.5% bovine serum albumin (BSA) for analysis and cell sorting with FACS Aria III (BD Biosciences).

### Cytokine measurement by CBA

Ischemic brains from vehicle- or MLC901-treated mice were homogenized in NP-40 containing buffer (10 mM Tris-HCL, ph 8, 150 mM NaCl, 1% NP-40, 10% glycerol, 5 mM EDTA and protease inhibitor cocktail (Roche Diagnostics)) as previously described^47^. Supernatants from brain homogenates or *ex vivo* cultured cells were harvested and the concentration of secreted cytokines (TNFα, IL1β, IL6 and CCL2) was detected using a CBA according to the manufacturer’s instructions (BD Biosciences). For comparison, data were normalized relative to the protein concentration of relative brain homogenate.

### Western blotting

As previously described^34^, brain tissues (vehicle- and MLC901-treated, sham-operated controls, n = 3 per group) were collected after 24 hours of reperfusion, and the fresh brains were cut into 2-mm-thick coronal sections 6 to 8 mm from the frontal pole, and carefully separated into ipsilateral and contralateral hemispheres, with respect to the infarct location. Samples were homogenized in four volumes of cold lysis buffer (20 nmol/l Tris pH: 7.5, 137 mmol/l NaCl, 2 mmol/l EDTA, 1% Triton X-100, 10% glycerol, and protease inhibitor cocktail) on ice. The homogenates were centrifuged at 12,000 *g* for 30 min at 4 °C. The supernatant was stored at −70 °C until further use. Protein concentrations were measured using conventional Bradford’s method. Fifty micrograms of proteins from each experimental group were applied to 10% SDS PAGE gels and electrophoresed for 1 h at 100 mA. Proteins were transferred onto a PVDF membrane in blotting buffer (156 mmol/l Tris, 1 mol/l glycine, PBS) for 90 min at 80 mA and blocked with 5% skim milk (Regilait) in PBS for 2 h at room temperature. The blotted membrane was then incubated overnight at 4 °C with the different primary antibodies: rabbit monoclonal phospho-NFκB p65 (Ser536) antibody (mAb#3033, diluted 1:1000; Cell Signal Technology) or rabbit monoclonal total NFκB p65 (D14E12) antibody (mAb#8242, diluted 1:1000; Cell Signal Technology) or rabbit polyclonal antibody against MMP9 (Millipore, AB 19026, 1/200). Western blots were incubated with horseradish-peroxidase conjugated anti-rabbit IgG (Jackson ImmunoResearch, diluted 1/15,000) for 1 h at room temperature. Specific bands were detected using the ECL Western blotting substrate and exposed to LAS-4000 image detection system. To control for sample loading, stripped membranes were rehybridized with a β-actin antibody (1:2000, Proteintech Group, USA) as internal control. Films with specific bands were scanned and quantified using an imaging densitometer. The optical densities of specific bands were analyzed with QUANTITY ONE software (Bio-Rad). Western blots were duplicated with 3 independent sets.

### Immunohistochemistry

Floating brain sections (25 μm thick) were immersed in 0.3% H_2_O_2_/PBS for 10 min, permeabilized in 0.1% Triton/PBS for 10 min and blocked with 3% goat serum/PBS for 2 h at room temperature as previously described^33^. After a PBS rinse, sections were incubated overnight with the following primary antibodies: rabbit polyclonal antibody against IL6 (Abcam, A6672, 1/500), GFAP (Dako, Z0334, 1/300) and MMP9 (Millipore, AB 19026, 1/200), mouse monoclonal antibody against Iba1 (Abcam 15690, 1/400), MPO (R&D, MAB3174) and rabbit monoclonal against phospho-NFκB p65 (Ser536) antibody (mAb#3033, diluted 1:1000; Cell Signal Technology), goat polyclonal antibody against TNFα (R&D, AF410—NA, 10μl/ml) and IL1β (R&D, AF401, 10μl/ml) and rabbit monoclonal antibody against Iba1 (Wako, 1/1000). After the primary incubation and three rinses in PBS, sections were then incubated in biotinylated horse anti-rabbit IgG (Jackson ImmunoResearch, diluted 1/15,000) for 2 h at room temperature. Protein expression was visualized by 3, 3-diaminobenzidine (DAB) staining using VectaStain ABC kit (Biovalley). All sections were washed and mounted with Entellan. Sections were observed using conventional microscopy. Signal specificity was assessed in negative control coverslips by omitting primary antibody. Images were acquired as single trans-cellular optical sections and averaged over at least four scans per frame. Analysis of the positive color intensity was performed by using the NIH Image J software (http://rsbwebnih.gov/ij), which allowed to measure the intensity levels of cells of each image saved as a 16-bit TIFF file. Five random and non-overlapping microscopic fields per section were examined at the cortical border of the positively stained area under 200X magnification in a blind fashion Results are given as mean of positive pixel ± SEM of three experiments.

### Statistical analysis

Statistical analysis was performed using Statistica software^TM^ and SigmaStat 2.3. Statistical values are presented as mean ± SEM. Significant differences between two groups of data (Vehicle group versus MLC901 group) were determined using a Mann & Whitney test for non-parametric data. For FACS experiment analyses, we chose to use a non-parametric permutation statistical test to overcome the variability of absolute values obtained between the replicates (3 independent experiments with 4 brains per experimental group). We used the non parametric Kruskal-Wallis one-way ANOVA test followed by Tukey’s multiple comparison to compare the five groups (Sham, Saline-MCAO NR, Saline-MCAO R, MLC901-MCAO NR and MLC901-MCAO R). In all analyses, the level of significance was set at *P* < 0.05.

## Data Availability

Materials, data and associated protocols are available to readers.
